# Widespread Alu repeat-driven expansion of consensus DR2 retinoic acid response elements during primate evolution

**DOI:** 10.1186/1471-2164-8-23

**Published:** 2007-01-19

**Authors:** David Laperriere, Tian-Tian Wang, John H White, Sylvie Mader

**Affiliations:** 1 Department of Biochemistry University of Montreal, Montreal, Québec, H3C 3J7, Canada; 2 Institute for Research in Immunology and Cancer, University of Montreal, Montréal, Québec, H3C 3J7, Canada; 3 Department of Physiology, McGill University, Montréal, Québec, H3G 1Y6, Canada; 4 Department of Medicine, McGill University, Montréal, Québec, H3G 1Y6, Canada

## Abstract

**Background:**

Nuclear receptors are hormone-regulated transcription factors whose signaling controls numerous aspects of development and physiology. Many receptors recognize DNA hormone response elements formed by direct repeats of RGKTCA motifs separated by 1 to 5 bp (DR1-DR5). Although many known such response elements are conserved in the mouse and human genomes, it is unclear to which extent transcriptional regulation by nuclear receptors has evolved specifically in primates.

**Results:**

We have mapped the positions of all consensus DR-type hormone response elements in the human genome, and found that DR2 motifs, recognized by retinoic acid receptors (RARs), are heavily overrepresented (108,582 elements). 90% of these are present in Alu repeats, which also contain lesser numbers of other consensus DRs, including 50% of consensus DR4 motifs. Few DR2s are in potentially mobile AluY elements and the vast majority are also present in chimp and macaque. 95.5% of Alu-DR2s are distributed throughout subclasses of AluS repeats, and arose largely through deamination of a methylated CpG dinucleotide in a non-consensus motif present in AluS sequences. We find that Alu-DR2 motifs are located adjacent to numerous known retinoic acid target genes, and show by chromatin immunoprecipitation assays in squamous carcinoma cells that several of these elements recruit RARs in vivo. These findings are supported by ChIP-on-chip data from retinoic acid-treated HL60 cells revealing RAR binding to several Alu-DR2 motifs.

**Conclusion:**

These data provide strong support for the notion that Alu-mediated expansion of DR elements contributed to the evolution of gene regulation by RARs and other nuclear receptors in primates and humans.

## Background

Alu repeats are SINEs (Short INterspersed Elements) whose original insertion in genomic sequences appears to have occurred shortly after the dawn of the primate lineage. While most SINEs arose from tRNA genes, Alu elements are derived from the 7SL RNA gene, which encodes a component of the protein signal recognition complex. The structure of modern Alu sequences arose from a duplication of primordial elements, which were approximately 200 bp in length and composed of an RNA polymerase III promoter at one extremity and a polyA tail at the other. Dimeric Alu elements expanded extensively throughout primate genomes as a result of a parasitic relationship with the transposition machinery encoded by L1 retrotransposons, which are LINEs (Long INterspersed Elements) [1,2], and today occupy ~10% of the human genome.

Several lines of evidence indicate that a variety of events associated with Alu transposition, recombination and expansion have contributed to genome evolution and to altering gene expression by several mechanisms. Because of their high CpG content and the tendency of CpG dinucleotides to mutate, and because of their prevalence, Alu repeats contain a substantial percentage of the single nucleotide polymorphisms in the human genome [1]. Their polyA tails act to seed microsatellite formation and expansion [3]. Moreover, Alu transposition has been proposed to underlie the expansion of segmental duplications that comprise approximately 5% of the human genome [4]. Conservative estimates suggest that 0.3–0.5% of human genetic disorders arise from mobile element insertion or from element-driven recombination events [5,6]. There is also evidence that Alu repeats inserted in an antisense orientation to gene transcripts can introduce alternative splicing sites [7,8], and that insertion of Alu sequences may alter epigenetic regulation of gene expression [9].

In addition to the above, increasing evidence suggests that Alu repeats are a source of elements that regulate transcriptional initiation by RNA polymerase II [10].

We have been interested in genome-wide mapping of hormone response elements in the promoter-proximal regions of human genes [11,12] to study their evolutionary conservation in the vicinity of genes, and their accessibility to cognate members of the nuclear receptor family. Nuclear receptors are the primary targets of a range of lipophilic signaling molecules such as steroid and thyroid hormones, vitamin D, retinoids, specific prostaglandins and cholesterol metabolites that regulate many aspects of physiology and metabolism, as well as embryonic development [13]. They directly regulate gene transcription by ligand-dependent recruitment of so-called coregulatory proteins that carry out the histone modifications, chromatin remodeling and binding of RNA polymerase II and ancillary factors necessary for initiation of transcription [13,14].

Nuclear receptors are composed of a series of domains, with the DNA binding and ligand binding domains being the most highly conserved. Most nuclear receptors function as homo- or heterodimers, and many receptor DNA binding domains recognize variants of consensus RGKTCA motifs arranged as either direct or inverted repeats[15]. Many non-steroid receptors recognize direct repeats in the form of heterodimers with members of the retinoid × receptor (RXR) family. For example, retinoic acid receptors (RARs) heterodimerized with RXRs specifically recognize retinoic acid response elements (RAREs) as RGKTCA direct repeats separated by 1, 2 or 5 bp (DR1, DR2 or DR5 motifs), whereas heterodimerized receptors for vitamin D (VDR) and thyroid hormone (TR) recognize DR3 and DR4 elements, respectively.

Evidence is accumulating that Alu repeats constitute a significant source of hormone response elements [16]. Notably, a motif, AGGTCAnnAGTTCG, found within most subclasses of AluS sequences, corresponds to a non-consensus DR2 element recognized by RARs, and has been shown to function as a RARE [17]. Signaling through RARs is of particular interest because retinoids control many aspects of embryonic development in a wide variety of organisms [13,18,19].

Here, we used a genome-wide screen to map DR-type hormone response elements in the human genome, and found that consensus DR2 elements are massively overrepresented relative to DR1 and DR5 RAREs and DRs recognized by other nuclear receptors, due to the presence of consensus DR2 motifs in >100,000 Alu repeats. The vast majority of these correspond to AluS sequences, which drove the Alu element expansion in primate that started 35–40 Myr ago. We also show that these "Alu-DR2" elements are present in retinoic acid regulated genes and bind RARs in vivo. Taken together, our data provide strong support for the notion that Alu-mediated expansion of DR elements contributed to the evolution of gene regulation by RARs and other nuclear receptors in primates and humans.

## Results

### Mapping DR-type hormone response elements in the human and mouse genomes

**Figure 1:**
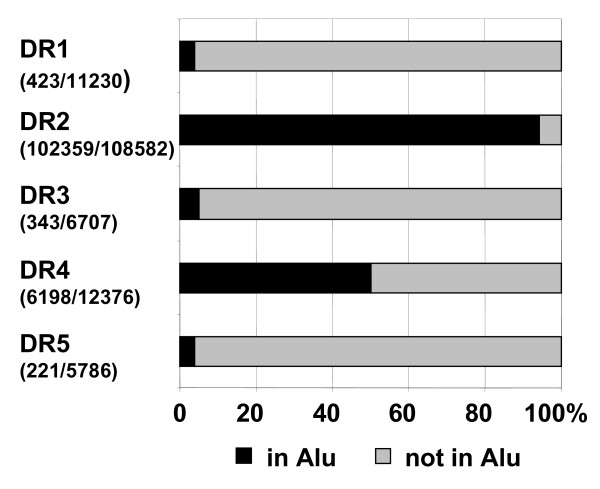
**Consensus DR1 to DR5 motifs embedded within Alu elements in the human genome**. The numbers below each DR correspond to the total number of repeats in Alu elements/total number of repeats in the genome.

A number of nuclear receptors recognize direct repeats of RGKTCA motifs spaced between 1 and 5 nucleotides as response elements, and individual receptors distinguish between DR elements based on inter-repeat spacings and subtle differences in motif sequence [15]. Sequencing of entire genomes has opened the way to mapping DNA response elements for nuclear hormone receptors on a genome-wide scale [11,12]. Given that mice have been used extensively as genetic models to study nuclear receptor signaling (e.g. [19]), we compared the frequency and distribution of consensus direct repeat response elements [RGKTCAn_i_RGKTCA, i = 1 to 5] both in the mouse and human genomes (Table 1). Although the frequency of these motifs should be comparable in random DNA sequences, our screen revealed that DR2 elements are about 10-fold more frequent than other DR sequences in the human genome, and are about 4-fold more common than in the mouse genome (108,582 vs 26,717). In addition, DR1 and DR4 elements occur more frequently than DR3 or DR5 elements (about 2-fold). To investigate the varying frequencies of different DR elements in the human and mouse genomes, we examined the proportions of each of these motifs found in transposable elements (TEs, Table 1). The vast majority of human DR2 motifs map within SINEs, in particular in Alu repeats (102,359/102,564 elements, see Figure 1 and Table 1), while approximately 50% of DR2 elements in the mouse (13,356) are found in retroviral LTRs, which only account for 1064 of the human elements (Table 1). DR4 motifs were also significantly associated within SINEs in the human genome, whereas DR1 motifs in TEs are found mostly in LINEs (Table 1). The frequencies of DR elements outside TEs is relatively similar, indicating that incorporation of DR elements within SINEs, LINEs or LTRs is the main cause for their variable distributions.

### Over 100,000 consensus DR elements are present in Alu repeats

To assess whether the large over-representation of DR2s in SINEs reflects amplification of a specific sequence, we calculated the frequencies of all motifs corresponding to the DR2 consensus. Of the 102,359 consensus DR2 elements located in Alu repeats, 92,686 corresponded to the sequence AGGTCAnnAGTTCA, and of these, AGGTCAggAGTTCA accounted for 82,319 occurrences, along with 9,877 copies with only one variation in the spacer nucleotides (data not shown). Other consensus DR2 elements containing one variation in the repeated motifs were much less frequent (less than 3,000 copies each for the single variations at the R and K positions, as calculated from the percentages in DR2 elements listed in Table 2). Consensus DR1 or DR3 motifs resulting from removal or addition of one nucleotide from the highly represented DR2 sequence were even more infrequent in Alu repeats (less than 0.2% or about 200 copies, Table 2, line 2).

The 102,359 DR2 elements, which are RAR target sequences, are by far the most frequent consensus DR motif present in Alu repeats (93.4%, Fig. 1), followed by DR4 elements, recognized specifically by TRs (5.7% or 6198 occurrences, Fig. 1). It is noteworthy that the 6198 Alu-DR4 elements represent ~50% of the total consensus DR4 elements in the genome (Fig. 1). Moreover, unlike in the DR2 consensus, RGGTCA motifs are found predominantly in the 3' repeat of the Alu DR4 sequences (Table 2, DR4 column). This position is bound by the TR when RXR-TR heterodimers bind to DR4 motifs. Since the TR has weak affinity for RGTTCA but recognizes RGGTCA motifs [20], these DR4 elements represent optimal TR binding sites.

### The vast majority of Alu-DR elements are present in AluS sequences

**Figure 2:**
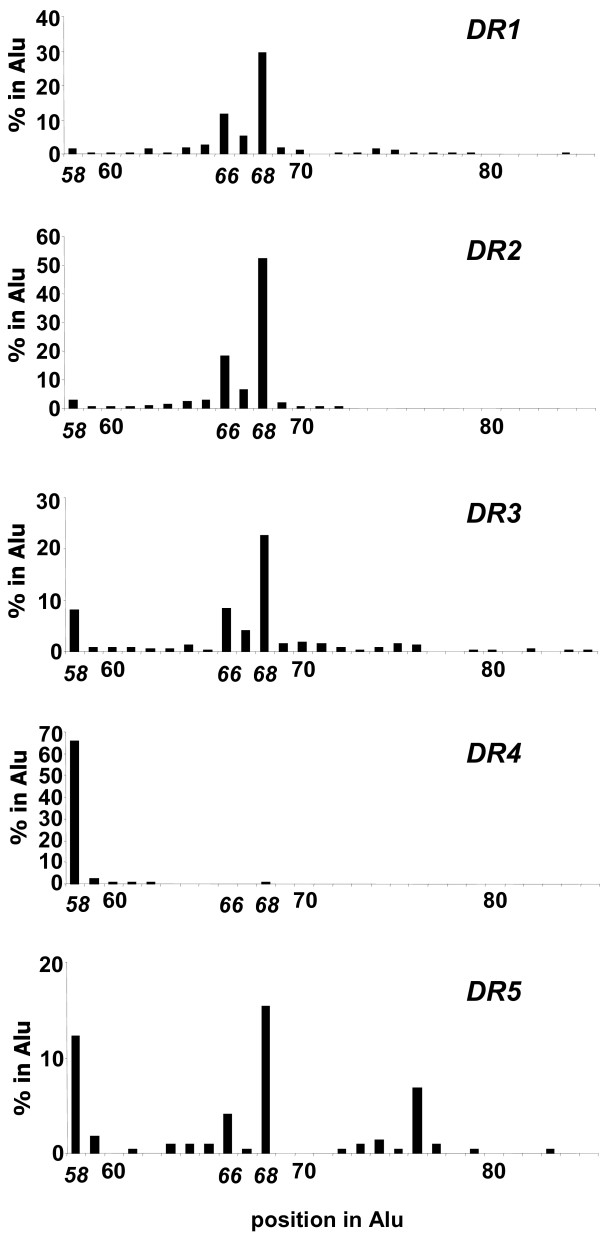
**Positions of DR motifs within Alu sequences in the human genome**. For clarity, only every 10th position is indicated, along with positions 58, 66 and 68. The positions indicated correspond to the 5'nucleotide of the 5'half site. Note the differences in scales of the ordinates.

Further analysis of human DR2-Alu elements revealed that their 5' ends are tightly clustered around positions 66–68 of the Alu repeat (Fig. 2). DR1 and DR3 elements showed largely similar distributions. In contrast, the 5' ends DR4 elements were found predominantly at position 58 of the repeat (Fig. 2). While DR5 elements were somewhat more widely distributed, most motifs started at positions 58, 66 or 68. The results reveal that DR2 and DR4 elements originated from partially overlapping sequences, where the 5' half-site of the consensus DR2 element corresponds to the 3' half-site of the DR4 motif (see below).

**Figure 3:**
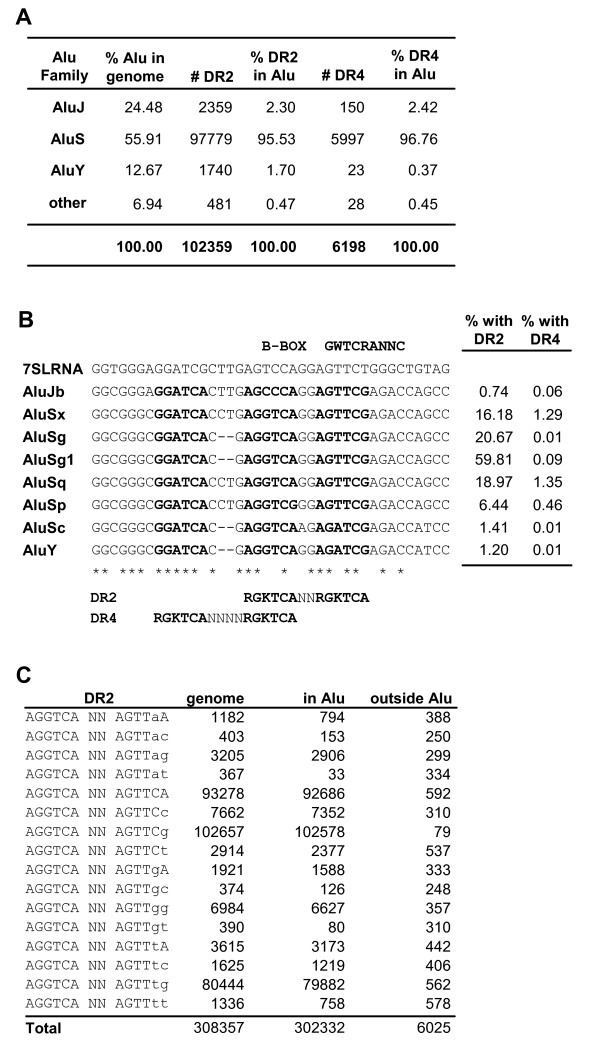
**Consensus DR2 and DR4 elements are found predominantly in AluS sequences**. (A) Distribution of consensus DR2 and DR4 elements within AluJ, S and Y families in the human genome. (B) Consensus sequences of AluJ, AluY and AluS subfamilies within the region containing the DR4 and DR2 element, and frequency of consensus DR2 RARE sequences within each subfamily. The position of box B of the polymerase III promoter is provided above. The sequences of the DR4 and DR2 consensus motifs are provided below. (C) Nucleotide substitution frequencies of the CpG dinucleotide present within AGGTCAnnAGGTCG motifs in AluS sequences.

The vast majority of consensus DR2 elements (97,779 or 95.5%; Fig. 3A) are present in AluS sequences, which represent just under 56% of total Alu sequences in the human genome. The overrepresentation of DR elements in AluS sequences, which largely drove the Alu repeat expansion 35–40 Myr ago [1,2,4], indicates that the majority of the consensus Alu-DR2 sequences (and related DR elements) present in the human genome multiplied through primate genomes during this AluS expansion. Consensus DR4 elements are similarly distributed primarily in AluS sequences (Fig. 3A). In contrast, consensus DR2 elements are markedly underrepresented in older AluJ and younger AluY sequences (2.3% and 1.7%, respectively; Fig. 3A). Of the 1.7% of DR2 motifs (1740 in total) present in potentially mobile AluY elements, only 41 motifs were not found to be present in the chimp and rhesus macaque genomes [see Additional file 1], indicating that more recent AluY-mediated transposition was not a major driving force in shaping retinoid-regulated gene expression in the human genome.

### Consensus Alu-DR2 elements arose predominantly via deamination of a methylated CpG dinucleotide

The distribution and frequency of DR2 elements in Alu sequences can be explained by the fact that the consensus DR2 RARE differs from the consensus sequence of several AluS subfamilies by a single G to A substitution in the 3'-most nucleotide of the DR2 repeat, whereas generation of an RARE consensus from Alu J or Y sequences would require multiple substitutions (Fig. 3B). Similarly, a single A to G or T substitution in the third position of the upstream GGATCA motif in AluS subfamilies Sx, Sq and Sp would generate a consensus DR4 element. Note however that although two different single mutations can lead to formation of a consensus DR4 versus one single replacement leading to a consensus DR2, the rate of conversion to a consensus DR2 is far superior to that to a DR4 element in all Alu subclasses.

It is noteworthy that generation of the DR2 RARE consensus could occur through deamination of a methylated C residue in the CpG dinucleotide of the AluS consensus, converting it to a T on the complementary strand of the direct repeat. A comparison of the frequencies of all four possible CpN dinucleotides in AGGTCAnnAGTTCN motifs present in AluS sequences indicates that CpA occurs far more frequently than CpT or CpC (Fig. 3C). Similarly, the conversion to T of the C residue of the CpG on the sense strand occurred far more frequently than other substitutions (Fig. 3C). Collectively, these data support the notion that the CpA dinucleotide in the DR2 element arose predominantly via a deamination-driven mechanism rather than by random base substitutions. The data are also consistent with other observations of high rates of CpG methylation [21] and of CpG dinucleotide decay generally in Alu repeats [22,23]. Further, the rate of conversion to consensus DR2s was similar in different subclasses of AluS elements differing by only one nucleotide (about 15–20% except for the AluSg1, a minor subfamily of AluSg [24], for which the rate of conversion is about 60%, Fig. 3B), suggesting generation of DR2s via the same methylation/deamination mechanism throughout evolution of the family. In addition, it is worth noting that the conversion to a perfect DR2 from the consensus AluS sequence, while introducing a change in the sequence of the box B of the RNA polIII promoter, occurs without altering its consensus (GWTCRANNC), suggesting that Alu elements containing perfect DR2 may still be mobile [25]. In a recent paper Price et al. defined over 200 AluS subfamilies [26], seven of which contain consensus DR2 elements. One of these, AluSg_14 shares the presence of the consensus RARE DR2 with three subfamilies [see Additional file 4]. These AluS subfamilies are likely examples of DR2 elements amplified by transposition instead of spontaneous mutation of a CpG.

### Distribution of Alu-DR2 elements relative to the 5'ends of human genes

**Figure 4:**
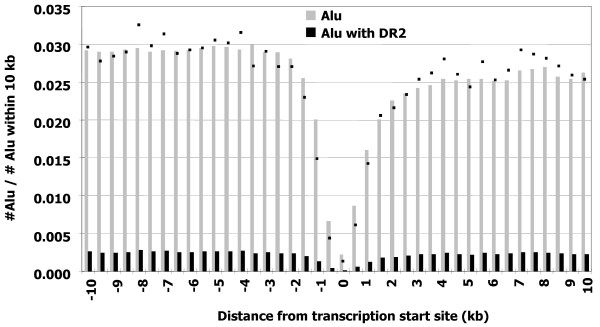
**Distribution of Alu sequences within the 10 kb flanking 5' ends of genes**. The 20 kb window for all genes annotated in the human genome were subdivided in 500 bp intervals. The number of Alu sequences and Alu sequences with a DR2 element were counted for each interval. Grey bars represent the ratio of the number Alu repeats within a given interval divided by the total number of Alu repeats within 10 kb of a gene, black bars represent the number DR2-containing Alu repeats within a given interval divided by the total number of Alu repeats within 10 kb of a gene, whereas black squares indicate the number Alu motifs with a DR2 element within a given interval divided by the total number of DR2-containing Alu repeats within 10 kb of a gene.

Generation of consensus DR2 in Alu elements via a mechanism implicating methylation of CpG dinucleotides, which is associated with transcriptional silencing, raises the question of whether these motifs can be recognized by RARs as functional binding sites. To investigate whether these elements could contribute to gene regulation, we first characterized the distribution of repeats containing DR2 elements relative to the 5' ends of human genes (Fig. 4). The distribution of Alu-DR2 differed from that of Alu repeats (χ^2 ^test, p-value = 2.84E-19), with a sharper reduction in the number of elements between -2 and +2 kb of transcriptional start sites. We note that there was a 20–30% further relative reduction in elements containing DR2 motifs within 1 kb of known 5' ends of genes compared to the general distribution of Alu elements, suggestive of a selection pressure against the presence of promoter-proximal Alu-DR2 sequences. However, RAREs are known to function as enhancer elements at distances up to 10–20 kb. Our mapping studies revealed over 18,000 Alu-DR2 elements lying within -10 kb and +10 kb of the 5'ends of >10,000 genes, [see Additional file 2], representing a substantial proportion of human genes.

### Association of RARs with Alu-DR2 elements *in vivo*

It is highly likely that most of the genes with proximal Alu-DR2 elements are not implicated in RAR-dependent gene regulation in a given cell type, with access to the sites being potentially limited by chromatin structure or binding of other proteins. For example, chromatin immunoprecipitation studies followed by microarray analysis (ChIP-on-chip) of genes regulated by related estrogen receptors in estrogen-sensitive breast carcinoma cells have suggested that only a minority of potential high affinity estrogen response elements of a given chromosome are accessible to receptors [27]. Moreover, as mentioned above, Alu repeat sequences are highly susceptible to methylation [21], which correlates with a closed chromatin structure and transcriptional silencing. However, 363 Alu-DR2 were found near 193 known retinoic acid target genes [see Additional file 2], suggesting that some Alu-DR2 elements can drive retinoid-regulated gene expression. We therefore tested the accessibility of several of these Alu-DR2 sequences to RARs by chromatin immunoprecipitation (ChIP) assay. We chose multiple elements present in the vicinity of 5 genes known to be RA-responsive in cells of squamous epithelial origin, *RAI1*, *GPRC5A *(*RAI3*), *SMYD5 *(*RAI15*), *RARRES1 *and *RARRES3*, and assessed RAR binding to these elements in SCC25 cells, a relatively well-differentiated human head and neck squamous cell carcinoma (HNSCC) line [28]. SCC25 cells express RARs and are retinoid-responsive [28], and are arrested by RA in the G0/G1 phase of the cell cycle [29]. The genes *RAI1 *and *GPRC5A *were originally identified as RA-regulated genes in HNSCC [30]. Similarly, *SMYD5 *and *RARRES 1 *and *3 *(retinoic acid receptor responder genes 1 and 3) were found to be induced by RA in human keratinocytes [31]. *RARRES3 *expression is lost in many tumors, and its product inhibits cell proliferation when transiently overexpressed [32].

**Figure 5:**
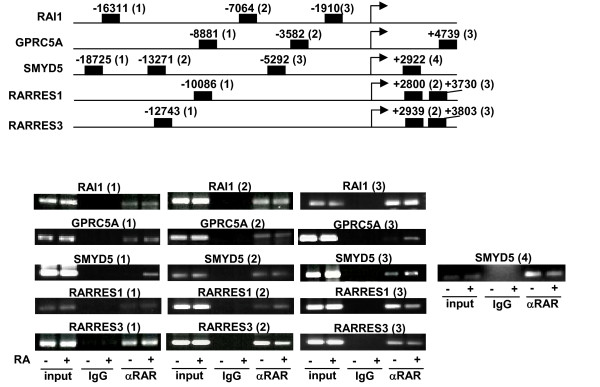
**RARs bind to Alu-DR2 motifs present in retinoic acid responsive genes in vivo**. Upper panel: Schematic representation of the promoter regions of selected known retinoic acid-responsive genes containing multiple consensus DR2 RAREs (AGGTCAggAGTTCA) embedded within an Alu repeat sequence. Lower Panel: Results of ChIP assays for RAR binding to promoter regions containing DR2 motifs. DNA from all immunoprecipitates (RAR-specific and control IgG) was amplified under similar conditions (40–42 cycles of PCR).

ChIP analysis with an anti-panRAR antibody revealed RAR binding to all elements tested (Fig. 5), whereas no RAR binding was observed to control sequences lacking DR2 elements (data not shown). In most instances, binding was either not RA-dependent or weakly enhanced by ligand, consistent with capacity of RARs to bind DNA in the absence of ligand [15]. However, immunoprecipitation of RARs associated with elements #3 in *GPRC5A *and #1 in *SMYD5 *was consistently ligand-dependent (Fig. 5, and data not shown). These studies are important as they confirm that all of the Alu-DR2 elements tested are in a chromatin environment that renders them accessible to RARs *in vivo*. We also confirmed that an oligonucleotide corresponding to the predominant DR2 element present in Alu repeats drove robust RA-inducible gene expression when cloned upstream of a truncated thymidine kinase promoter (data not shown), supporting its potential to function as an RARE *in vivo*.

In addition, we obtained further evidence for the function of Alu-DR2 elements as RA-dependent enhancer sequences from the publicly-available results of the ENCODE Project Consortium, which mapped transcription factor binding sites including those for RARs in 44 selected regions of the human genome (30 Mb in total) [33]. ChIP-on-chip studies of these regions using retinoic acid-treated human HL60 cells identified binding regions for RARα, 17 of which encompass Alu elements containing perfect DR2 motifs (Table 3). Of these, we note that expression of caveolin-1, the product of the *CAV1 *gene, was found to be upregulated in retinoic acid-treated HL60 cells [34]. Taken together, the data of Fig. 5 and Table 3 strongly support the capacity of Alu-DR2 sequences to function as RAREs.

## Discussion

Nuclear receptor signaling, particularly that of retinoic acid receptors, has been widely studied in animal models, most notably the mouse. Our findings reveal striking differences in hormone response element frequency and distribution between rodent, and primate and human genomes. Results of the genome-wide mapping of high affinity hormone response elements for members of the nuclear receptor family of transcription factors presented herein indicate that consensus DR2 retinoic acid response elements are vastly overrepresented relative to other DR response elements in the human genome, and are enriched approximately 4-fold in the human genome relative to the mouse. This enrichment arose as a result of the presence of DR2 motifs in Alu repeats, which are primate-specific. Movement of Alu repeats and other classes of transposable elements has been recognized as a potential mechanism for introduction of RNA polymerase II transcriptional regulatory elements for a variety of transcription factors into promoter regions of genes [10,35,36], thus altering the signal transduction pathways regulating specific genes. For example, a recent study [36] used DNA-binding profiles of representatives of different classes of transcription factors from the TRANSFAC database [37] to identify transcription factor binding sites in transposable elements located in promoter regions. This study did not screen for RAREs, for which matrices are not provided in the TRANSFAC database.

While the presence of imperfect RAREs in Alu elements and their capacity to function as RAREs in reporter assays has been reported previously [17], the main findings from our study are the unexpected large number of consensus DR2 RAREs, and the demonstration that several of these sites are functional RAR binding sites in vivo. It is striking that the genome-wide number of imperfect DR2s corresponding to the AGGTCAnnAGTTCG consensus, which matches the consensus sequences of most AluS subclasses, is not much higher (102,657) than that of the consensus DR2s (93,278), underlining the unexpectedly high number of perfect DR2 RAREs in the human genome (Fig. 3C). The generation of consensus DR2 elements by deamination of the CpG dinucleotide within the 3' AGTTCG motif is supported by the comparably high number of AGGTCAnnAGTTTG sequences (80,444), which diverge from a perfect DR2 more than the motif found in consensus AluS sequences but represent the other possible site generated by methylation/deamination at the CpG dinucleotide in the B box of Alu repeats.

Demonstration that Alu DR2 RAREs are functional binding sites in their normal chromatin context is crucial given the high frequency of methylation of Alu sequences in the human genome [21], and the association between DNA methylation and silencing of transcription in general, and of Alu repeats specifically [38]. For instance, expression and retinoic acid responsiveness of one of the genes studied above, RARRES1, is frequently inhibited in malignancies by hypermethylation [39], and the extent of methylation is inversely correlated with the state of differentiation of malignant cells [40].

The capacity of Alu-DR2 motifs to function as RAREs is supported by several lines of evidence. We identified 363 motifs within 10 kb of the 5' ends of 193 known retinoic acid-regulated genes [see Additional file 2]. It is also noteworthy that 8 Alu-DR2 within 10 kb of 9 genes overlap with DNase I-hypersensitive sites from primary human CD4+ T cells [41] [see Additional file 2]. We confirmed the capacity of several Alu-DR2 elements to bind RARs in vivo in retinoic acid-sensitive SCC25 cells by chromatin immunoprecipitation analysis of receptor binding to sequences located proximal to five genes known to be retinoic acid-responsive in squamous carcinoma cells. These studies indicate that access of RARs to the DR2 motifs under study was not impeded by their presence in an Alu repeats in these cells. Moreover, our analyses of ChIP-on-chip studies from the ENCODE consortium showed that several Alu-DR2 elements in human HL60 cells are sites of RA-dependent recruitment of RARs (Table 3), further supporting the function of Alu-DR2 elements as RAREs.

We found that DR2 consensus elements were present in substantial numbers in multiple AluS subfamilies, indicating that they arose throughout the course of AluS expansion. The perfect RARE DR2 motif corresponds to the consensus sequence in seven divergent AluS subfamilies defined by Price et al. [26], in addition to being found sporadically throughout numerous other AluS subfamily sequences (Additional file 4 and unpublished results). It should be noted that the downstream half-site of the DR2 element lies within box B of the Alu RNA polymerase III promoter, but that the conversion of RGTTCG to RGTTCA to generate the consensus DR2 motif does not necessarily impair its competence for retrotransposition, consistent with transcriptional activity of Alu RNA polymerase III promoters containing RGTTCAANNC box B motifs [25,42].

Consistent with their presence predominantly in AluS sequences, which represent relatively ancient Alu elements, we found that the vast majority of human Alu-DR2 motifs are present in the chimp and macaque genomes. A few Alu-DR2 elements were apparently unique to the human genome, although in some cases they corresponded to regions that were not sequenced in the simian genomes. While none of these uniquely human Alu-DR2s are adjacent to known retinoid-regulated genes in humans, it will be of interest in the future to characterize the potential of RA to regulate their expression in appropriate cell model systems. It is also noteworthy that one of these genes, CYP2A6, was shown to be regulated by the nuclear receptor hepatic nuclear factor 4 (HNF4) [43], which, like RARs, can recognize DR2 motifs [44].

Our previous work identified a consensus DR3 element recognized by the VDR in vitro and in vivo in the proximal promoter region of the cathelicidin antimicrobial peptide gene [45], which was found to be strongly regulated by vitamin D in a variety of human cell types. However, neither the regulation nor the response element was conserved in mouse, and studies by others [46] showed the DR3 to be conserved in primates, but not other species, and present in an Alu repeat. This represents a clear example of an Alu-driven divergence in nuclear receptor gene regulation between primates/humans and other species. Results presented here demonstrate that Alu repeat expansion similarly has led to the generation of functional binding sites for retinoic acid receptors. Further studies of DR2-containing Alu repeats will be necessary to determine precisely to which extent these elements have contributed to the primate-specific regulation of target genes by retinoic acid.

## Conclusion

We find that consensus DR2 motifs are heavily overrepresented in the human genome relative to other DR response elements due to their presence in a subset of Alu motifs, in particular in AluS sequences. Although DR4s are found far less frequently in Alu repeats, those present account for 50% of consensus DR4 motifs in the human genome. Consensus Alu-DR2 elements arose predominantly through deamination of a methylated CpG dinucleotide present in AluS elements rather than through random base substitutions. While Alu elements can be transcriptionally repressed by methylation-driven mechanisms, our screen identified Alu-DR2 elements in promoter proximal regions of numerous retinoid regulated genes. Importantly, we found that Alu-DR2s are accessible to RARs in vivo in two RA-responsive human cell lines, indicating that the expansion of DR2 motifs within Alu elements during the course of primate evolution contributed to altering regulation of gene expression by retinoic acid.

## Methods

### Genome-wide identification of DR1-DR5 within Alu sequences

The human (hg17, May 2004) and mouse (mm6, March 2005) reference sequence chromosomes from the UCSC Genome Browser database [47,48] were scanned for RGKTCA motifs separated by 1 to 5 bp (DR1-DR5) using a previously described program perl program [11,12]. The RefSeq Genes track [49], downloaded September 21 2005, was used to identify the DR2 located -10 to +10 kb of gene 5'-ends. A custom SQL program used the chromosomal location of Alu sequences from the RepeatMasker track [50] to identify included DR1-DR5. The same program used the chromosomal location of regions bound by RARα from the ENCODE Affy Sites track to identify DR2 elements within these regions. Another SQL program was used to identify the DR2 in AluY that are not included within Human/Chimp (hg17 vs panTro1) and Human/Rhesus (hg17 vs rheMac2) pairwise BLASTZ alignment [51]. The χ^2 ^test to compare the distribution of Alu-DR2 to Alu repeat was carried out with the chisq.test function of the R statistical computing environment [52]. The cladogram of AluSg subfamilies was generated with PHYLIP 3.6 [53] using the consensus of AluS families from Repbase [54] and AluS subfamilies with RARE DR2 in their consensus from Price et al [26]. All programs were run on the bioinformatics cluster of The Quebec Bioinformatics Network (BioneQ) and are available upon request.

### ChIP assays

Approximately 2 × 10^7 ^SCC25 cells were treated for 1 h with RA 10^-6^M or vehicle. Then cells were rinsed with PBS and incubated for 10 min in PBS containing 1% formaldehyde at 37°C. Cells were washed with ice-cold PBS and the cell pellets were resuspended in 300 μl of lysis buffer (1% SDS, 10 mM EDTA, 50 mM Tris-HCl (pH 8.1) and 1× protease inhibitor cocktail (Roche Diagnostics, Laval, Que, Canada). Sonication was performed by pulsing three times for 15 s to reduce DNA to fragments of ~300–500 bp in length. Centrifugation was then performed to obtain cleared cell lysates. Lysates were diluted 1:10 in ChIP dilution buffer (1% Triton X-100, 1 mM EDTA, 150 mM NaCl and 20 mM Tris-HCl pH 8.1). A portion of the lysate was taken as INPUT control sample. Pre-clearing was done by incubating the cell lysates with salmon sperm DNA (1 μg/ml), BSA (1 μg/ml) and protein A-agarose slurry (Santa Cruz Biotechnology, Santa Cruz CA) for 1 h. Cell lysates were then incubated by overnight rotation at 4°C with 4 μg of either normal rabbit IgG or primary antibody, followed by rotation-incubation with protein A-agarose for 2 h. The beads were rinsed three times sequentially with TSE I buffer (0.1% SDS; 1% Triton X-100; 2 mM EDTA; 150 mM NaCl; 20 mM Tris-HCl, pH 8.1), TSE II buffer (0.1% SDS; 1% Triton X-100; 2 mM EDTA; 500 mM NaCl; 20 mM Tris-HCl, pH 8.1), LiCl Wash Buffer (0.25 M LiCl; 1% NP-40; 1% deoxychlolate (Na salt); 1 mM EDTA; 10 mM Tris-HCl, pH 8.1) and 2 times TE buffer (10 mM Tris-HCl; 1 mM EDTA, pH 8.0). The beads were eluted twice with 250 μl elution buffer (1% SDS; 0.1 M NaHCO_3_). To reverse the cross-linking, the eluates were incubated in a 65°C incubator overnight and then the samples were purified with PCR purification kit (Qiagen, Mississauga, Ont, Canada). PCR primers used for ChIP assays are described in Additional file 3.

## List of abbreviations

ChIP, chromatin immunoprecipitation; DR, direct repeat; HNF4, hepatic nuclear factor 4; LINEs, long interspersed elements; RA, retinoic acid; RAR, retinoic acid receptor; RARE, retinoic acid response element; RXR, retinoid × receptor; SINEs, Short INterspersed Elements; TR, thyroid hormone receptor; VDR vitamin D receptor.

## Authors' contributions

DL was responsible for the genome-wide analysis of response element distribution in the human and mouse genomes, TTW performed the ChIP assays, JW and SM conceived the study, directed DL and TTW in the analysis of their work and wrote the article. All authors read and approved the final manuscript.

## Supplementary Material

Additional File 1 A table of DR2 embedded in Alu found in Human but not Chimp and Rhesus genome. Click here for file

Additional File 4 AluS subfamilies with RARE DR2 in their consensus. Click here for file

Additional File 2 A table of all DR2 in Alu within 10 kb of human gene 5' ends. Click here for file

Additional File 3 ChIP Primers for genes with DR2-Alu. Click here for file

## Figures and Tables

**Table 1:** Consensus RGKTCA direct repeats found in the human and mouse genomes.

	**Total**		**outside transposable elements**		**in SINEs**		**in LINEs**		**in LTRs**	
	Human	Mouse	Human	Mouse	Human	Mouse	Human	Mouse	Human	Mouse
**DR1**	11230	16250	5950	9741	687	3679	3633	788	960	2042
**DR2**	108582	26717	4186	5623	102564	7157	768	581	1064	13356
**DR3**	6707	6336	3935	4192	997	571	936	605	839	968
**DR4**	12376	8893	4397	5549	6602	638	621	457	756	2249
**DR5**	5786	8846	4337	5338	430	1059	552	1073	467	1376

**Table 2:** RGKTCA direct repeats found in Alu elements.

**DR sequence**	**# in Alu**	**% in DR1**	**% in DR2**	**% in DR3**	**% in DR4**	**% in DR5**
AGGTCA AGGTCA	2888	0.90	49.90	0.73	47.64	0.83
AGGTCA AGTTCA	93067	0.19	99.59	0.11	0.06	0.05
AGGTCA GGGTCA	78	23.08	39.74	6.41	25.64	5.13
AGGTCA GGTTCA	2212	2.40	96.11	1.04	0.31	0.14
AGTTCA AGGTCA	524	0.95	46.95	1.72	49.62	0.76
AGTTCA AGTTCA	2389	1.21	95.10	1.80	0.63	1.26
AGTTCA GGGTCA	10	20.00	60.00	0.00	20.00	0.00
AGTTCA GGTTCA	137	12.41	78.10	3.65	2.19	3.65
GGGTCA AGGTCA	5244	0.08	29.67	0.27	69.68	0.30
GGGTCA AGTTCA	1285	0.70	91.91	0.47	6.69	0.23
GGGTCA GGGTCA	94	1.06	27.66	9.57	54.26	7.45
GGGTCA GGTTCA	44	11.36	68.18	2.27	13.64	4.55
GGTTCA AGGTCA	1096	0.18	42.43	0.27	55.84	1.28
GGTTCA AGTTCA	197	5.58	41.11	27.92	13.71	11.68
GGTTCA GGGTCA	21	9.52	61.91	0.00	19.05	9.52
GGTTCA GGTTCA	258	24.03	35.66	18.61	7.75	13.95
RGKTCA RGKTCA	109544	0.39	93.44	0.31	5.66	0.20

**Table 3:** Alu-DR2 elements within ENCODE ChIP-on-chip RARα binding regions.

**DR2 position**	**RARα binding region**	**region length**	**DR2 position in region**	**nearest gene**	**distance (kb)**
chr11:5234577–5234590	chr11:5234153–5234769	617	437	*HBG2*	-2.0
chr11:116438036–116438049	chr11:116437268–116438894	1627	768	*KIAA0999*	36.1
chr11:5214973–5214986	chr11:5214632–5215086	455	354	*HBD*	-2.5
chr15:41723046–41723059	chr15:41722559–41723965	1407	500	*CATSPER2*	5.2
chr15:41832787–41832800	chr15:41832276–41832882	607	524	*PDIA3*	6.9
chr15:41893221–41893234	chr15:41892245–41893398	1154	989	*MFAP1*	11.0
chr19:59507050–59507063	chr19:59506478–59507251	774	572	*LILRA5*	9.1
chr20:33783677–33783690	chr20:33783609–33783958	350	68	*RBM39 (RNPC2)*	9.9
chr20:33795208–33795221	chr20:33795121–33795786	666	87	*RBM39 (RNPC2)*	-1.6
chr21:33890599–33890612	chr21:33890070–33890930	861	529	*DONSON*	-7.7
chr21:39545848–39545861	chr21:39545687–39546093	407	161	*BRWD1*	61.5
chr22:30446428–30446441	chr22:30446058–30446529	472	383	*C22orf30 (MGC50372)*	-13.1
chr6:74021882–74021895	chr6:74021526–74021987	462	369	*C6orf148*	7.7
chr6:108492975–108492988	chr6:108492625–108493122	498	363	*OSTM1*	9.6
chr7:115763054–115763067	chr7:115762699–115763680	982	368	*CAV1*	4.2
chr7:116952987–116953000	chr7:116952795–116953258	464	192	*CTTNBP2*	154.5
chrX:122568351–122568364	chrX:122567587–122568522	936	777	*THOC2*	-12.1
