# Supplementary material for: Widespread Alu repeat-driven expansion of consensus DR2 retinoic acid response elements during primate evolution
Source: BMC Genomics. 2007 Jan 19;8:23. doi: 10.1186/1471-2164-8-23 (PMC1785376; doi:10.1186/1471-2164-8-23)
Supplement: Additional File 4 — AluS subfamilies with RARE DR2 in their consensus. [file 1471-2164-8-23-S4.pdf]

A

AluSx   61 TCACCTGAGGTCAGGAGTTCGAGACCAGCCTGGCCAACATGGTGAAACCCCGT 113
AluSx_4 61 TCACCTG**AGGTCA**GG**AGTTCA**AGACCAGCCTGGCCAACATGGTGAAACCCCGT 113
AluSg   61 TCAC--GAGGTCAGGAGTTCGAGACCAGCCTGGCCAACATGGTGAAACCCCGT 111
AluSg1   61 TCAC--GAGGTCAGGAGTTCGAGACCAGCCTGGCCAAGATGGTGAAACCCCGT 111
AluSg_4 61 TCAC--G**AGGTCA**GG**AGTTCA**AGACCAGCCTGGCCAAGATGGTGAAACCCCGT 111
AluSg_14 61 TCAC--G**AGGTCA**GG**AGTTCA**AGACCAGCCTGGCCAAGATGGTGAAACCCCGT 111
AluSg_16 61 TCAC--G**AGGTCA**GG**AGTTCA**AGACCAGCCTGGCCAAGATGGTGAAACCCCGT 111
AluSg_24 61 TCAC--G**AGGTCA**GG**AGTTCA**AGACCAGCCTGGCCAACATGGTGAAACCCCGT 111
AluSg_27 61 TCAC--G**AGGTCA**GG**AGTTCA**AGACCAGCCTGGCCAAGATGGTGAAACCCCGT 111
AluSq   61 TCACCTGAGGTCAGGAGTTCGAGACCAGCCTGGCCAACATGGTGAAACCCCGT 113
AluSq_6 61 TCACCTG**AGGTCA**GA**AGTTCA**AGACCAGCCTGGTCAACATGGTGAAACCCCGT 113
AluSp   61 TCACCTGAGGTCGGGAGTTCGAGACCAGCCTGACCAACATGGAGAAACCCCGT 113
AluSc   61 TCAC--GAGGTCAAGAGATCGAGACCATCCTGGCCAACATGGTGAAACCCCGT 111
RGKTCANNRGKTCA

B

Alu Family	# in genome	% with DR2	Repbase Alu family
AluSx_4	485	59.59	AluSx (75%)
AluSg_4	4712	67.32	AluSg1 (76%)
AluSg_14	3587	58.49	AluSg (75%)
AluSg_16	995	68.84	AluSg1 (67%)
AluSg_24	1979	62.81	AluSg (82%)
AluSg_27	300	60.33	AluSg1 (71%)
AluSq_6	1032	59.88	AluSq (85%)

C

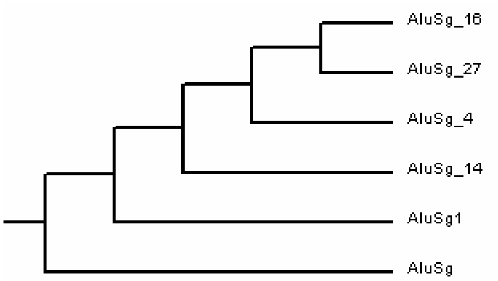

Supplemental Figure 1 - (A) Alignment of AluS with Alu families from Price et al. [26] that contain consensus RARE DR2. (B) Frequency of RARE DR2 for Alu families from Price et al. (C) Cladogram of AluSg subfamilies
