# Supplementary material for: Widespread Alu repeat-driven expansion of consensus DR2 retinoic acid response elements during primate evolution
Source: BMC Genomics. 2007 Jan 19;8:23. doi: 10.1186/1471-2164-8-23 (PMC1785376; doi:10.1186/1471-2164-8-23)
Supplement: Additional File 3 — ChIP Primers for genes with DR2-Alu. [file 1471-2164-8-23-S3.pdf]

ChIP Primers for genes with DR2-Alu

	Gene			Name of the primer
1.	***RAI1***	5′	5′-gcttctggcagtacagccca	RAI1D1ChIP5′
		3′	5′-cttccgcttcagcctcccta	RAI1D1ChIP3′
		5′	5′-catgatggctcaccactgt	RAI1D2ChIP5′
		3′	5′-ctaagtgcaactccagcctc	RAI1D2ChIP3′
		5′	5′-actttgggagtccgaggcag	RAI1D3ChIP5′
		3′	5′-cctggagtgcaatggtgcga	RAI1D3ChIP3′
**2.**	***SMYD5***	5′	5′-gttgacagcccaagtgaccc	SMYD5D1ChIP5′
		3′	5′-caggcgcggttatcccagca	SMYD5D1ChIP3′
		5′	5′-tgcggtggcttacgcctgta	SMYD5D2ChIP5′
		3′	5′-ggctggagtgcaatggcact	SMYD5D2ChIP3′
		5′	5′-agtagatgtggccaggtgc	SMYD5D3ChIP5′
		3′	5′-gttgcccaagctggagtgca	SMYD5D3ChIP3′
		5′	5′-tcagccttccgagtagctgg	SMYD5D4ChIP5′
		3′	5′-gcgcagtggctcatgcttgt	SMYD5D4ChIP3′
**3.**	***GPRC5A***	5′	5′-agatatcaggccaggcacgg	GPRC5AD1ChIP5′
		3′	5′-tacaggactgcaccaccagg	GPRC5AD1ChIP3′
		5′	5′-ttacaggcatgcaccaccac	GPRC5AD2ChIP5′
		3′	5′-tcctctgaagggaatgtccg	GPRC5AD2ChIP3′
		5′	5′-ggctcacacctgtaatcccagca	GPRC5AD3ChIP5′
		3′	5′-ggagtgcagtggcgtgatct	GPRC5AD3ChIP3′
4.	***RARRES1***	5′	5′-gagcagctgggattccaggt	RARRES1D1ChIP5′
		3′	5′-ttgaggccaggcatgatggc	RARRES1D1ChIP3′
		5′	5′-ggattacaggtgcctgccac	RARRES1D2ChIP5′
		3′	5′-cggtggctcacgcctgtaat	RARRES1D2ChIP3′
		5′	5′-gctcactgcaacctctgcct	RARRES1D3ChIP5′
		3′	5′-ggctctcgcctgtaatccca	RARRES1D3ChIP3′
5.	***RARRES3***	5′	5′-ggctagagtgcagtggcatg	RARRES3D1ChIP5′
		3′	5′-gtgcagtggctgacacctgt	RARRES3D1ChIP3′
		5′	5′-cacggtggctcatgcctgta	RARRES3D2ChIP5′
		3′	5′-tgcaatggcacgatctcggc	RARRES3D2ChIP3′
		5′	5′-aggcacagtggctcactcct	RARRES3D3ChIP5′
		3′	5′-tgtctcctaggctggagtgc	RARRES3D3ChIP3′
